# Interaction behavior between coarse-particle pyrite and fine-particle pyrite in flotation

**DOI:** 10.1038/s41598-025-06128-0

**Published:** 2025-07-02

**Authors:** Xianchen Wang, Hong Li

**Affiliations:** 1 School of Resources and Environmental Engineering, Moutai Institute, Renhuai, 564507 China; 2 Engineering Research Center of Phosphorus Resources Development and Utilization of Ministry of Education, Wuhan, 430073 China

**Keywords:** Gold-bearing pyrite, Coarse particles, Fine particles, Flotation, Interaction behavior, Chemical engineering, Surface chemistry, Environmental chemistry

## Abstract

Pyrite is commonly associated with gold deposits and serves as a gold-bearing mineral. The flotation recovery of gold-bearing pyrite generally determines the overall gold recovery. In this study, pyrite from a gold ore in southwestern Guizhou Province was selected to investigate the interaction behavior between coarse-particle pyrite (CPy) and fine-particle pyrite (FPy) during flotation. Flotation tests were conducted in combination with laser particle size analysis, optical microscopy, scanning electron microscopy (SEM), energy-dispersive spectrometry (EDS), and a self-assembled “Particle Attachment Dynamic Observation System.” The results showed that the recovery of FPy (P80 = 26.75 µm) was lower than that of CPy (P80 = 147.89 µm) under the same collector concentration (butyl xanthate). However, the flotation recovery of the CPy-FPy mixture was consistently higher than that of either CPy or FPy alone, particularly at low collector concentrations. Further investigation confirmed that CPy acted as a carrier for FPy, enhancing its recovery into the froth during mixed flotation. Calculations based on the extended Derjaguin–Landau–Verwey–Overbeek (EDLVO) model demonstrated that butyl xanthate increases hydrophobic forces between pyrite particles, promoting agglomeration. This finding explains why the flotation recovery of the CPy-FPy mixture in a butyl xanthate suspension exceeds that of CPy or FPy alone.

**Supplementary Information:**

The online version contains supplementary material available at 10.1038/s41598-025-06128-0.

## Introduction

Pyrite (FeS_2_) is one of the most widely distributed sulfide minerals in the Earth’s crust, commonly found in various rocks and ore deposits, especially in fine- particle disseminated gold deposits^1,2^. Actually, gold is closely related to pyrite, which not only coexists with gold but also often serves as its primary carrier mineral^3,4^. In Guizhou Province, China, pyrite is the main gold-bearing mineral in many fine-particle disseminated gold deposits^5^. A large number of studies have shown that most of the gold element in the fine-particled disseminated gold ores in Guizhou province exists in pyrite and other sulfides, while only a small amount is hosted in other minerals^6–8^. For instance, one study revealed that in a fine-particle disseminated gold sampled from Guizhou^9^, 94.88% of the gold exists as encapsulated gold, while free-milling gold and intergrown gold account for only 5.12% of the total gold content. The gold predominantly occurs as lattice-bound gold and nano-sized inclusions within pyrite, displaying an uneven and sparsely disseminated distribution. Conventional methods such as crushing, grinding, and mineral processing struggle to directly liberate the gold. Therefore, a common processing strategy is to first recover gold-bearing pyrite, followed by gold extraction from the pyrite itself^10^. In this context, the recovery efficiency of pyrite directly influences the overall gold recovery.

**Fig. 1:**
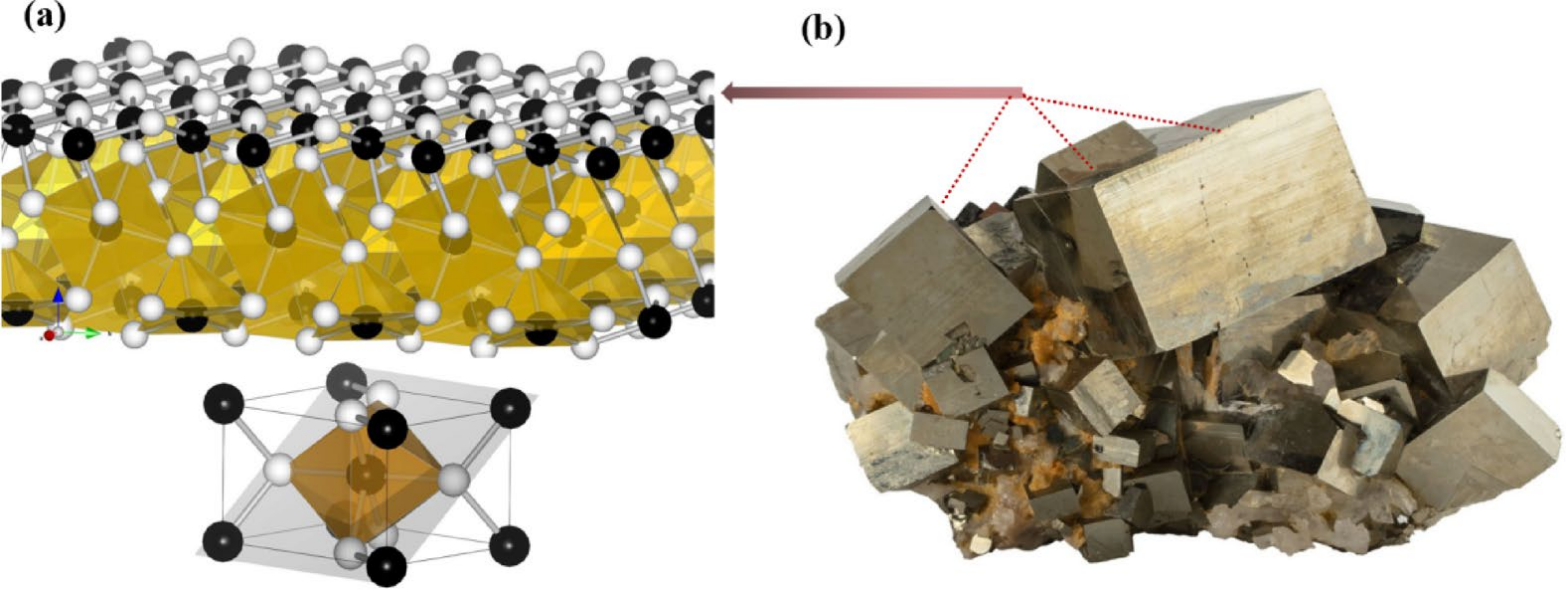
Unit cell structure and picture of pyrite (a: unit cell structure; b: pyrite picture; black spheres are Fe atoms and white spheres are S atoms).

Pyrite may occur in polymetallic ores or exist independently as the dominant sulfide mineral^11,12^. Its crystal structure and morphology are illustrated in Fig. 1^13^. Pyrite belongs to the isometric system with a NaCl-type crystal structure and the *Pa3* space group^14,15^. Each unit cell contains 4 Fe atoms and 8 S atoms in the crystal structure of pyrite^13^. This structure results in a large number of Fe-S covalent bonds and definite equilibrium defects on the surface of pyrite.Additionally, adsorption of water molecules on the (100) surface of pyrite is most stable when oxygen is adsorbed onto Fe sites, exerting a slight oxidation effect on adjacent S atoms. This contributes to pyrite’s inherent hydrophobicity^16^. Although the basic crystal structure remains consistent, variations in the metallogenic environment can result in differences in pyrite morphology, particle size, and surface reactivity. As a result, its hydrophobic properties may differ from one deposit to another^17^. Nevertheless, pyrite generally exhibits strong hydrophobicity under the influence of flotation collectors and is commonly recovered by flotation techniques^18^.

Flotation is a commonly used mineral processing method. Approximately 90% of non-ferrous metals and 50% of ferrous metals are processed using flotation worldwide^19^. In the flotation processing, particles in water interact with bubbles and selectively attach to bubbles according to their relative hydrophobicity, floating onto the liquid level as foams^20,21^. And the hydrophilic particles remain in the pulp (a suspension of water and mineral particles) and are separated as tail products^22^. Particle size plays a crucial role in flotation performance. The flotation efficiency of mineral particles is higher when the particle size is 10–150 µm^23^. For ultrafine particles, low collision probability with bubbles–due to their small mass and tendency to follow fluid streamlines–leads to poor attachment^24,25^. Conversely, overly coarse particles (e.g., > 0.1 mm) are prone to detachment due to their weight, reducing flotation efficiency^26^, which makes it difficult to attach to the bubbles, resulting in the reduction of flotation efficiency and recovery. The particle size range of different minerals may vary and depends on a number of factors, such as independent testing or continuous testing, or laboratory scale and industrial scale^26^.

Grinding is essential for liberating valuable minerals from gangue prior to flotation^27^. When the target minerals are fine-particle, overgrinding may occur, producing excessive fine particles^28,29^. Pyrite in gold ore has a wide range of particle size, leading to uneven particle sizes post-grinding. Even in conventional flotation, both fine particles and coarse particles exist in the pulp^30^. This size disparity influences flotation behavior and may result in significant pyrite losses, especially when fine particles dominate. For example, Sun et al.^31^ reported that approximately 71% of the gold in Huilong gold ores (Guizhou) was hosted in pyrite, arsenopyrite, and other sulfides. When ground to 96% passing − 0.075 mm, the gold concentrate achieved a grade of 25.14 g/t and a recovery of 86.94%. Similarly, Faraz et al.^32^ investigated flotation of gold ore with pyrite as the main gold carrier. Under a grinding particle size of K80 at 146 µm, the gold recovery rate after 60 min of flotation was the highest, reaching 90.6%. These studies suggest that flotation recovery of gold-bearing pyrite remains suboptimal. The presence of fine particles is a major limiting factor. Indeed, fine particle flotation is one of the persistent challenges in mineral processing, particularly when the valuable mineral content is low^24,33^. The common methods of flotation for fine minerals, such as shear flocculation and oil agglomeration method, cannot get good results. One of the key reasons is the relatively low mass fraction of gold-bearing pyrite compared to the surrounding gangue, which hampers flotation efficiency for fine particles.

Many studies have addressed pyrite flotation in gold ores, focusing on reagent selection, process optimization, mineral interactions, and crystal structure analysis^34–36^. However, the interaction between coarse-particle pyrite (CPy) and fine-particle pyrite (FPy) during the flotation has not been thoroughly investigated. Understanding these interactions is critical for optimizing flotation performance, guiding reagent selection, and improving overall gold recovery. In this study, pyrite from a gold deposit in southwestern Guizhou Province was used to explore the flotation behavior and interaction between CPy and FPy. The findings aim to contribute to the optimization of gold-bearing pyrite flotation in the region and offer insights applicable to similar ores elsewhere.

## Materials and methods

### Materials

**Fig. 2:**
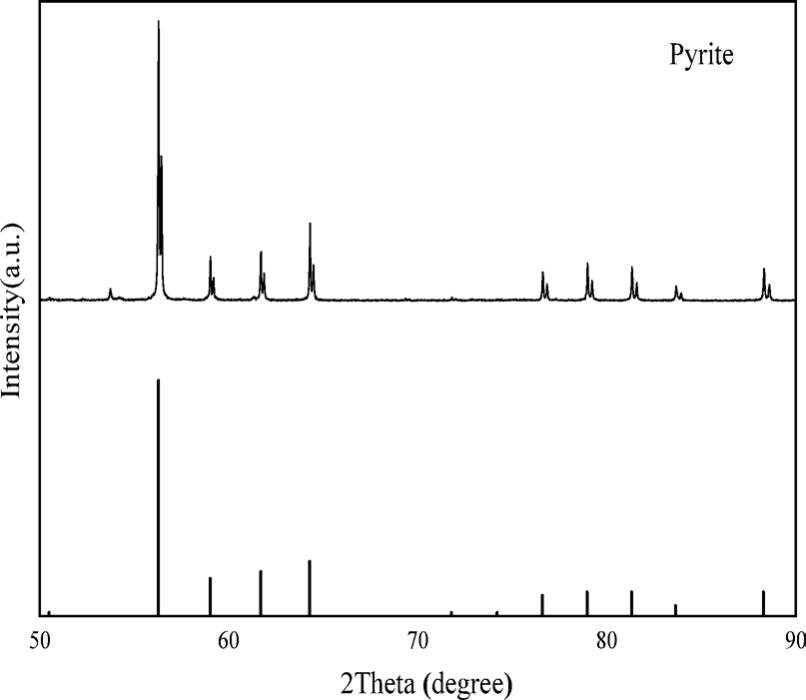
X-ray diffraction pattern of this pyrite sample.

**Fig. 3:**
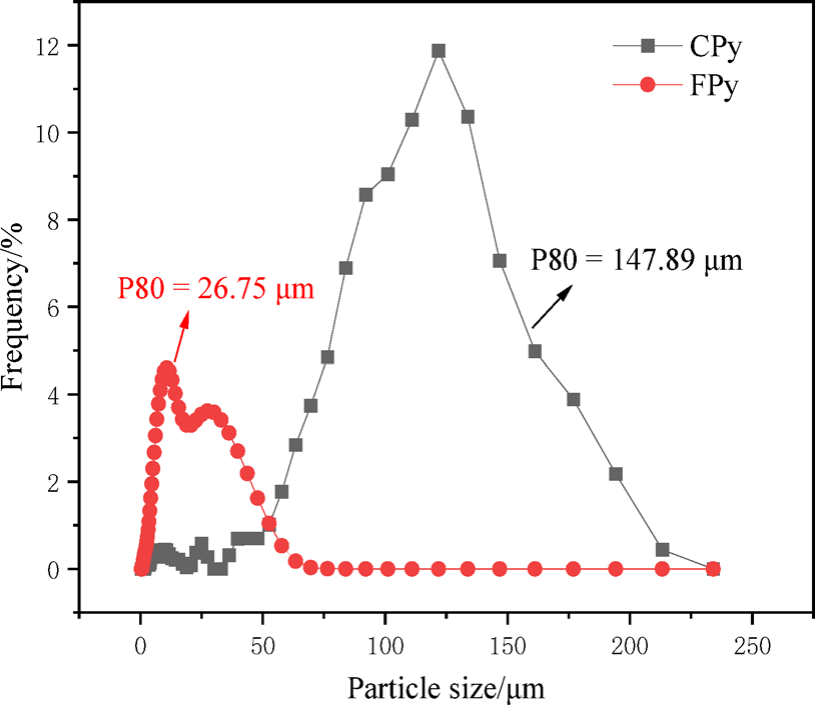
Original particle size distribution of CPy and FPy samples.

The gold ore sample was obtained from a gold mine in southwest of Guizhou Province, China. Block samples rich in pyrite were selected and subsequently crushed for further processing. CPys were initially separated manually, and then FPys were separated using a shaking table. The pyrite was finely ground using an agate grinder and screened through − 140 mesh (0.109 mm) and 200 mesh (0.075 mm) standard screens. The fraction between − 0.109 mm and + 0.075 mm was collected, sealed in a ziplock bag, and designated as CPy. The fraction below 0.075 mm was further ground for 0.5 h and stored in a ziplock bag as FPy. The X-ray diffraction (XRD) pattern of the sample closely matched the standard pyrite pattern (see Fig. 2), showing no significant peaks of other minerals, indicating that the sample was predominantly composed of pyrite^37^. The chemical composition of the pyrite was analyzed using an X-ray fluorescence (XRF) spectrometer (Panalytical Zetium, Malvern Panalytical, Netherlands), and the results are summarized in Table 1. The analysis showed that Fe and S were the main elements in the samples, while the concentrations of impurity elements were very low. The particle size distributions of CPy and FPy were measured using a laser particle size analyzer (Bettersize 2600, Dandong Bettersize Instruments Co., Ltd., China), as illustrated in Fig. 3. The P80 values for CPy and FPy were 147.89 µm and 26.75 µm, indicating that CPy particles were significantly larger than FPy particles. Terpenic oil (2# oil) was used as the frother, hydrochloric acid (HCl) and sodium hydroxide (NaOH) were employed as pH modifiers, and butyl xanthate (C_4_H_9_OCSSNa) was selected as the collector. It should be noted that the 2^#^ oil was a technical reagent (TR) with relatively lower purity, while HCl, NaOH, and C_4_H_9_OCSSNa were analytical-grade reagents (AR) with higher purity. All experiments were conducted using deionized water with a resistivity of approximately 18 MΩ cm.

**Table 1:** The chemical composition of this pyrite.

Element	Fe_2_O_3_	SO_3_	CaO	SiO_2_
Content/%	69.89	28.77	0.74	0.57

### Methods

#### Microflotation tests

The microflotation test was carried out in the XFGC II flotation machine (XFGCII5 ~ 35, Jilin Exploration Machinery Plant, China) with a 40 mL cell. Precisely 2.0 g of CPy, FPy, or a mixture of CPy and FPy were accurately weighed and added to 40 mL water^38^, followed by ultrasonic pretreatment for 10 min. The ultrasonic treatment sample was transferred into 40.0 mL flotation cell of flotation machine with deionized water. After stirring for 1 min, HCl (0.1 mol/L) or NaOH (0.1 mol/L) solution was added to adjust the pulp pH for 2 min, ensuring a final pH of 5 or 9^39^, so that the pH of pulp was 5 or 9. Add the collector butyl xanthate for stirring 2 min, add 2 drops of foaming reagent 2^#^ oil for stirring 2 min, followed by flotation for 1 min. The froth and tailing products were collected, filtered, dried, and weighed to calculate flotation recovery^40^.

#### Optical microscope observation

The optical microscope (BX51-P, Olympus, Japan) was employed to observe the particle morphology in the pulp containing CPy, FPy, or the mixture of CPy and FPy (CPy: FPy = 1:1) under different conditions. The pulp preparation followed procedures similar to the microflotation tests. Two to three drops of the pulp suspension were placed on a glass slide. Then the particle distribution and particle morphology in the pulp were observed and captured pictures.

#### Mineral particle size analysis

The particle size distribution was tested by a laser particle size analyzer (Bettersize 2600, Dandong Baxter Instrument Co. LTD, China) which can detect apparent size changes and provide information on interparticle aggregation behavior. During the testing, an appropriate amount of CPy, FPy, or the mixture of CPy and FPy (CPy: FPy = 1:1) was respectively mixed with 40 mL of deionized water, followed by ultrasonication for 10 min. Then the pH of the pulp was adjusted to 5 or 9 with HCl and NaOH, respectively. Flotation reagents butyl xanthate were added and allowed to react fully for 2 min. Then all the pulp was poured into the test cell to measure and obtain the information of the particle size. Each test was repeated three times, and average values were reported.

#### SEM observation

Scanning electron microscopy (SEM) (CLARA, TESCAN, Czech Republic) was used to observe FPy attached to the CPy surface. The sample preparation involved accurately weighing and mixing CPy and FPy, followed by ultrasonic treatment for 10 min. The mixture was then immersed in solutions with different conditions. After stirring for 5 min, the CPy was slowly removed from the middle of the suspension by using a sample spoon, dried and sprayed with gold. Observation was performed at 500 magnification, and then representative CPy particle was observed at 2000, 3000 magnification, respectively. Elemental composition of the surface was analyzed using energy-dispersive spectrometry (EDS; Xplore 30, Oxford Instruments, UK) at 3000 magnification. Due to the large volume of samples, only 500 and representative 3000 magnification images were selected for publication.

#### Dynamic observation of particle attachment

In this study, a particle adhesion dynamic observation system was used to observe the attachment of FPy to CPy. Approximately 0.5 g of mineral sample was ultrasonically treated and transferred to the sample cell. The magnetic stirrer was activated for 1 min, after which HCl or sodium NaOH solutions (both at 0.1 mol/L) were added to adjust the pulp pH for 2 min, as required. Subsequently, the collector butyl xanthate was introduced, and the mixture was stirred for an additional 2 min before stopping. A plane of massive pyrite was sonicated, adhered to the sample fixing rod with glue, and immersed in the aqueous suspension. The vertical position of the pyrite in the suspension was kept consistent throughout all tests. The rotor speed of the magnetic stirrer was set to 250 rpm in the experiment, and the attachment of particles to the surface of the massive pyrite was monitored at 10 s, 30 s, 60 s, 120 s, and 180 s intervals. Images were captured after the suspension settled. The images at the 180 s mark were selected as representative.

#### Interaction energy calculation

The Derjaguin–Landau–Verwey–Overbeek (DLVO) theory was put forward by Derjaguin, Landau, Verwey, and Overbeek in the 1940s to explain the interaction between particles and their aggregation in water^41^. DLVO theory mainly considers two interacting forces: van der Waals forces and Electrostatic force. Nevertheless, in addition to van der Waals and Electrostatic force, there are also hydrophobic forces in mineral flotation system, which could also affect the particle condensation and dispersion behavior. Therefore, the interaction forces between particles in this paper were calculated using the extended DLVO theory (EDLVO theory)^42^. For the interaction force between pyrite particles in the flotation system in the presence of flotation reagents, the total energy (V_TED_) can be expressed as follows in Eq. (1):

1 $${V}_{TED}={V}_{WA}+{V}_{ER}+{V}_{HA}$$

where V_DT_ is the sum of interaction forces between particles; V_WA_ is the van der Waals interaction force; V_ER_ is the electrostatic interaction force; V_HA_ represents hydrophobic or hydration interaction.

1. Van der Waals interaction force *V*_WA_

For coarse–fine particles systems with large particle size differences, the ball-plate interaction model is adopted, and the formula can be expressed as Eq. (2).

2 $$V_{WA} = \frac{{ - A_{{12\left( {13} \right)}} }}{6H} \times \frac{{R_{1} R_{2} }}{{R_{1} + R_{2} }}$$

where H is the interaction distance (nm) between particles, R_1_ and R_2_ are the radius (nm) of CPy (56.05 um) and FPy (6.65 um), respectively. A_12(13)_ is the interacting Hamaker constant for the mineral particle 1 in medium 2 (medium 3), can be calculated by Eq. (3).

3 $${A}_{12(13)}={\left(\sqrt{{A}_{1}}-\sqrt{{A}_{2(3)}}\right)}^{2}$$

The Hamaker constant of pyrite, butyl xanthine, solution and water in vacuum are shown in Table 2. According to Eq. (3), A_12_ = 1.51 × 10^–20^ and A_12_ = 2.37 × 10^–20^.

**Table 2:** Hamaker constant of pyrite, butyl xanthate solution and water interaction in vacuum^43,44^.

	A_1_/Pyrite	A_2_/Butyl xanthate	A_3_/Water
Hamaker constant	12.0 × 10^–20^	5.0 × 10^–20^	3.7 × 10^–20^

The van der Waals interaction force between mineral particles with similar particle sizes is considered as the force between two spheres, and the Equation can be expressed as:

4 $$V_{WA} = \frac{{ - A_{{12\left( {13} \right)}} R_{1} }}{6H}$$

(2) Electrostatic interaction force V_ER_

For coarse–fine particles systems with large particle size differences, the ball-plate interaction model is adopted, and the formula can be expressed as Eq. (5).

5 $${V}_{ER}=\frac{\pi \varepsilon {R}_{1}{R}_{2}}{{R}_{1}+{R}_{2}}\left({\varphi }_{1}^{2}+{\varphi }_{2}^{2}\right)\left[\frac{2{\varphi }_{1}{\varphi }_{2}}{{\varphi }_{1}^{2}+{\varphi }_{2}^{2}}p+q\right]$$

6 $$p=\text{ln}\left[\frac{1+\text{exp}(-kH)}{1-\text{exp}(-kH)}\right]$$

7 $$\text{q}=\text{ln}[1-\text{exp}(-2kH)]$$

where ε = ε_r_ × ε_0_, ε_0_ is the absolute permittivity in vacuum 8.854 × 10^–12^ C^−2^/(J m), ε_r_ is the relative permittivity of the dispersed medium, ε_r_ is the relative permittivity of the medium (78.5 C^−2^/(J m for water and 6.95 × 10^–10^ C^−2^/(J m) for butyl xanthate solution) and^45,46^. φ_1_ and φ_2_ are the surface potentials (substituted by Zeta potential) of the different mineral particles, respectively, mV. The k is the Debye length (thickness of the double electric layer), selected 0.104 nm^−1^^47^.

For the systems with similar particle sizes, it can be calculated by the interaction model between spheres, expressed as Eq. (8):

8 $${V}_{ER}=4\pi {\varepsilon }_{r}{\varepsilon }_{0}{R}_{1}{\varphi }_{0}^{2}\text{ln}[1+\text{exp}(-kH)]$$

(3) Polar Interfacial Interaction V_HA_

For systems with similar particle size, the interaction model with the ball is selected, expressed as Eq. (9):

9 $${V}_{HR}=2\pi {R}_{1}{h}_{0}{V}_{H}^{0}\text{exp}\left(\frac{{H}_{0}-H}{{H}_{0}}\right)$$

For coarse–fine particle systems with large particle size differences, the ball-plate interaction model is used, and the formula can be expressed as Eq. (10):

10 $${V}_{HA}=2\pi \frac{{R}_{1}{R}_{2}}{{R}_{1}+{R}_{2}}{h}_{0}{V}_{H}^{0}\text{exp}\left(\frac{{H}_{0}-H}{{H}_{0}}\right)$$

where *H* is the interaction distance (nm); *H*_0_ is the Equilibrium contact distance between two particle surfaces, which is approximately 0.2 nm^48^. h_0_ is the attenuation length, which is approximately 1 nm^49^.

${V}_{H}^{0}$ is the polar interaction energy constant, calculated as Eq. (11):

11 $${V}_{H}^{0}=2\left[\sqrt{{\gamma }_{3}^{+}}\left(\sqrt{{\gamma }_{1}^{-}}+\sqrt{{\gamma }_{2}^{-}}-\sqrt{{\gamma }_{3}^{-}}\right)+\sqrt{{\gamma }_{3}^{-}}\left(\sqrt{{\gamma }_{1}^{+}}+\sqrt{{\gamma }_{2}^{+}}-\sqrt{{\gamma }_{3}^{+}}\right)-\sqrt{{\gamma }_{1}^{+}{\gamma }_{2}^{-}}-\sqrt{{\gamma }_{1}^{-}{\gamma }_{2}^{+}}\right]$$

where ${\gamma }_{1}^{+},{\gamma }_{2}^{+},{\gamma }_{3}^{+}$ denote the electron acceptor components of the surface energy of particle 1, particle 2, and medium 3, respectively. ${\gamma }_{1}^{-}{,\gamma }_{2}^{-},{\gamma }_{3}^{-}$ represents the electron donor component of the surface energy of particle 1, particle 2, and medium 3, respectively.

For the water medium, ${\gamma }_{3}^{+}={\gamma }_{3}^{-}=$ 25.5 mJ/m^2^, ${\gamma }_{1}^{+},{\gamma }_{2}^{+}{,\gamma }_{1}^{-}{,\gamma }_{2}^{-}$ can be obtained from Eq. (12):

12 $$\left( {1 + \cos \theta } \right)\gamma_{L} = 2\left( {\sqrt {\gamma_{s}^{d} \gamma_{L}^{d} } + \sqrt {\gamma_{s}^{ + } \gamma_{L}^{ - } } + \sqrt {\gamma_{s}^{ - } \gamma_{L}^{ + } } } \right)$$

where ${\upgamma }_{\text{L}}$ is the surface energy of liquid, ${\upgamma }_{\text{L}}^{\text{d}}$ is the dispersive component of liquid surface energy, ${\upgamma }_{\text{L}}^{+}$ is the electron acceptor component of liquid surface energy, ${\upgamma }_{\text{L}}^{-}$ is the electron donor component of liquid surface energy. For the water medium, ${\upgamma }_{\text{L}}=$ 72.8 mJ/m^2^, ${\upgamma }_{\text{L}}^{\text{d}}$=$21.8\text{ mJ}/{\text{m}}^{2}$. ${\upgamma }_{\text{s}}^{\text{d}}$ is the dispersion component of the solid surface energy, ${\upgamma }_{\text{s}}^{+}$ is the electron acceptor component of the solid surface energy, ${\upgamma }_{\text{s}}^{-}$ is the electron donor component of the solid surface energy, and θ is the contact Angle of the liquid on the solid surface. These parameters of pyrite used in the calculation are shown in Table 3. For pyrite, ${\gamma }_{s}^{+}$ is approximately zero. Equation (12) can be simplified as Eq. (13):

13 $$\left(1+\text{cos}\theta \right){\gamma }_{L}=2\left(\sqrt{{\gamma }_{s}^{d}{\gamma }_{L}^{d}}+\sqrt{{\gamma }_{s}^{-}{\gamma }_{L}^{+}}\right)$$

${\gamma }_{s}^{d}$ can be obtained from the Eq. (14), expressed as follow:

**Table 3:** The parameters of pyrite used in the calculation^43^.

pH = 9						pH = 5					
Water			Butyl xanthate			Water			Butyl xanthate		
${\gamma }_{s}^{d}$	${\gamma }_{s}^{-}$	θ	${\gamma }_{s}^{d}$	${\gamma }_{s}^{-}$	θ	${\gamma }_{s}^{d}$	${\gamma }_{s}^{-}$	θ	${\gamma }_{s}^{d}$	${\gamma }_{s}^{-}$	θ
79.47	25.79	32°	79.47	10.26	54°	79.47	10.68	53.36°	79.47	0.71	74.9°

14 $$A=1.51\times {10}^{-21}{\gamma }_{s}^{d}$$

## Results and discussions

### Flotation

#### Effect of collector concentration and pulp pH on pyrite flotation

**Fig. 4:**
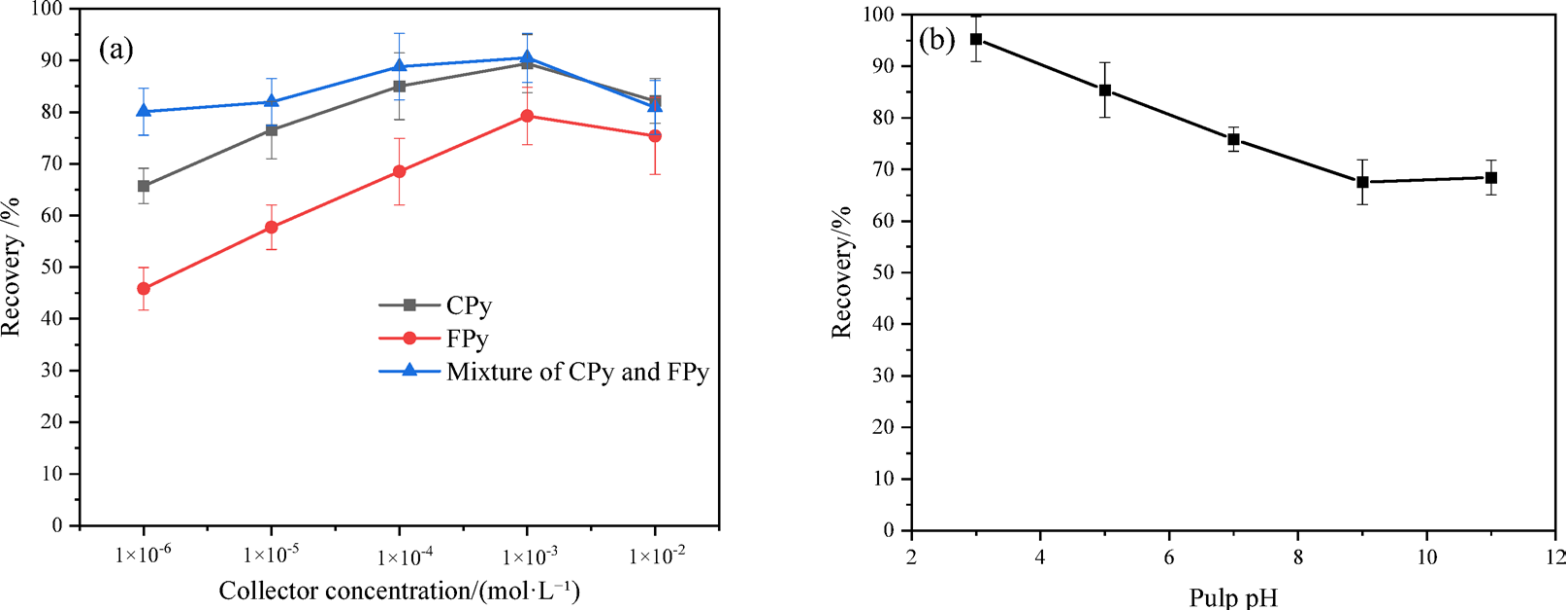
Effect of collector concentration and pulp pH on pyrite flotation (**a**): collector concentration; (**b**): pulp pH.

The flotation performance of pyrite is strongly influenced by its crystal structure, which is in turn affected by the ore-forming environment. As a result, the floatability of pyrite varies significantly across different deposits. In this study, butyl xanthate–one of the most commonly used collectors for pyrite–was selected to investigate the effect of collector concentration on the flotation recovery of CPy, FPy, and their mixture without adding pH modifiers, as illustrated in Fig. 4a. With increasing butyl xanthate concentration, the flotation recovery of CPy, FPy, and their mixture exhibited a rising trend, followed by a plateau, indicating saturation of collector adsorption sites. The optimal flotation recovery of pyrite was attained at a butyl xanthate concentration of 1 × 10⁻^3^ mol/L. Notably, the flotation recovery of FPy was lower than that of CPy at the same collector concentration, consistent with the flotation characteristics of fine minerals. Fine particles, due to their low inertia and small mass, tend to follow streamlines around air bubbles, leading to a lower collision and attachment probability compared to coarse particles. Importantly, the flotation recovery of the mixture of CPy and FPy was consistently higher at each collector concentration compared to that of CPy and FPy alone. This suggests an interaction between CPy and FPy that enhances overall pyrite flotation recovery. It is speculated that the adsorption of FPy onto the surface of CPy facilitates carrier flotation, thereby improving the flotation recovery of FPy and, in turn, the overall flotation recovery. This hypothesis will be further investigated in future studies.

Pulp pH is known to have a pronounced effect on pyrite flotation^50–52^. In order to study the effect of pulp pH on the flotation recovery of gold-bearing pyrite, the CPy flotation recovery at different pH was tested in the presence of 1 × 10^–3^ mol/L butyl xanthate, and the results were shown in Fig. 4b. As pH increased, the flotation recovery of CPy decreased and eventually leveled off, which is consistent with previous studies^53,54^. This trend can be attributed mainly to the formation of surface oxide films. Under acidic conditions, these oxide films are decomposed, exposing fresh pyrite surfaces that readily interact with the collector. Nevertheless, ferrous ions were hydrolyzed to ferrous hydroxide in alkaline solutions (Eq. (15)), which was subsequently oxidized to iron hydroxide (Eq. (16)). Therefore, the reduced floatability of pyrite was related to the coverage of hydrophilic iron hydroxide on the pyrite surface, which was not conducive to the collection of butyl xanthine.

15 $${Fe}^{2+}+2{H}_{2}O\to Fe(OH{)}_{2}{+2H}^{+}$$

16 $${Fe(OH{)}_{2}+OH}^{-}\leftrightarrow Fe(OH{)}_{3}{+e}^{-}$$

Although pyrite shows poor floatability under alkaline conditions, it retains good floatability in neutral to mildly acidic media. However, since acidic environments can corrode iron-based flotation equipment, industrial pyrite flotation is typically conducted under slightly alkaline conditions.

#### Effect of CPy on flotation of FPy

To further elucidate the interactive behavior between CPy and FPy, flotation tests were carried out to evaluate how CPy affects the floatability of FPy. The mass ratio of CPy and FPy in the mixture was 1:1. The theoretical flotation recovery of the mixture of CPy and FPy was defined as ε_T_, which was the weighted value of flotation recovery of CPy and FPy without interference from each other. Meanwhile, the actual flotation recovery (ε_F_) of the mixture of CPy and FPy under different collector concentrations was used to compare. The theoretical flotation recovery ε_T_ was calculated by Eq. (17):

17 $${\upvarepsilon }_{T}={\upgamma }_{1}{\upvarepsilon }_{1}+{\upgamma }_{2}{\upvarepsilon }_{2}$$

where ε_1_ and γ_1_ were the flotation recovery of FPy without CPy and the mass fraction of FPy in the mixture of f CPy and FPy, respectively. ε_2_ and γ_2_ were the flotation recovery of CPy without FPy and the mass fraction of CPy in the mixture of f CPy and FPy, respectively. It was worth noting that γ_1_ + γ_2_ = 1.

**Fig. 5:**
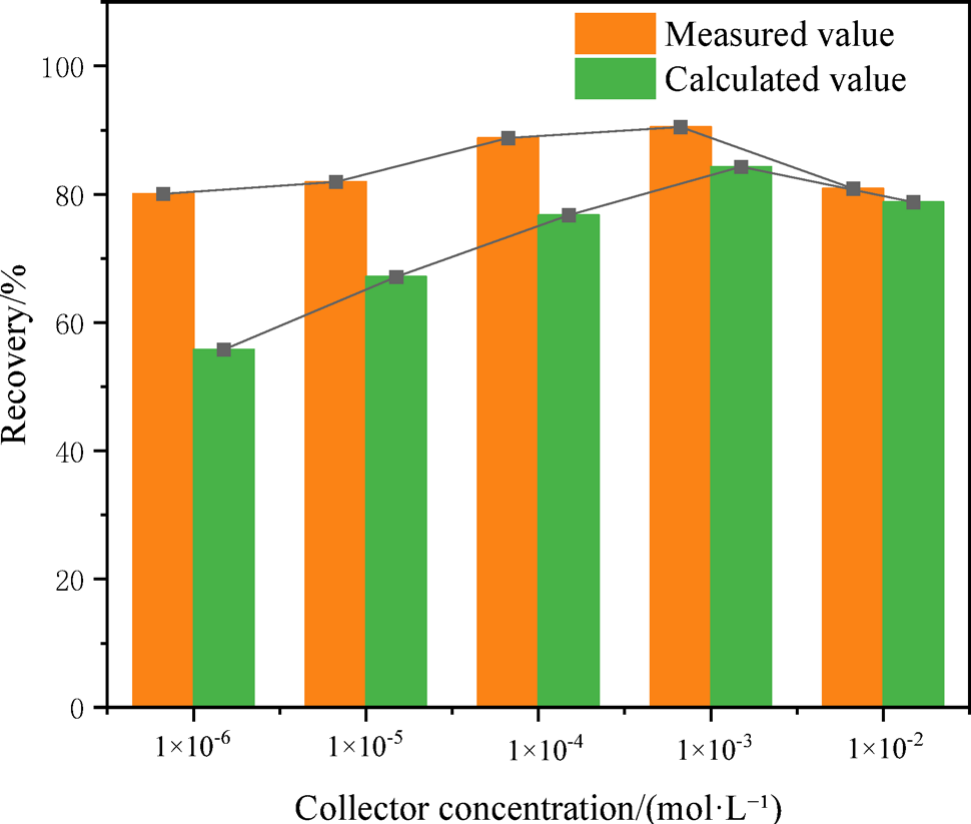
Theoretical flotation recovery (ε_T_) and actual flotation recovery (ε_F_) of the mixture of CPy and FPy.

The ε_F_ and ε_T_ of the mixture of CPy and FPy were shown in Fig. 5. It could be clearly seen that the ε_F_ of the mixture of CPy and FPy was bigger than ε_T_ at each collector concentration. At a butyl xanthate concentration of 1 × 10^–6^ mol/L, the actual flotation recovery ε_F_ of the CPy–FPy mixture reached 80.09%, significantly exceeding the theoretical recovery ε_T_ of 55.77%, which indicated that there was an interaction between CPy and FPy, and promoted the increase of the total pyrite flotation recovery, especially at the low butyl xanthate concentrations. The difference of ε_F_ and ε_T_ of the mixture of CPy and FPy was very small when the amount of butyl xanthate was 1 × 10^–2^ mol/L. Nonetheless, an interaction between CPy and FPy occurred, and this interaction was beneficial for flotation and worthy for further investigation.

The collision frequency f_c_[s^−1^ m^−3^] between fine-particle and coarse-particle in case of turbulent conditions, which could be expressed as follows:

18 $$f_{c} \propto \left( {D_{f} + D_{c} } \right)^{3} n_{f} n_{c}$$

19 $$f_{c} \propto D_{c}^{3} n_{f} n_{c} \left( {If\;Df \ll Dc} \right)$$

20 $${n}_{c}=\frac{{m}_{c}/{\rho }_{c}}{{V}_{c}}=\frac{{m}_{c}/{\rho }_{c}}{{\Phi }_{c}{ {D}_{c}}^{3}}$$

21 $$f_{c} \propto \frac{{m_{c} }}{{\rho_{c} \Phi_{c} }}n_{f} \left( {{\text{When}}\;{\text{particle}}\;{\text{was}}\;{\text{sphere}}} \right)$$

where D_f_ and D_c_ are the diameters of fine and coarse particles, respectively, and n_f_ and n_c_ are the concentrations of fine and coarse particles in the suspension^55^, m_c_, ρ_c_ and V_c_ are the mass and density of the coarse particles and the volume of the coarse particle group, respectively. These equations indicate that, under turbulent conditions, the particle–particle collision frequency is more strongly influenced by particle mass and concentration than by particle size. Therefore, the recovery of pyrite in flotation pulp increases with the number of pyrite particles^56^.

### Verification of FPy adhesion to CPy

#### Particle size analysis of mixture of CPy and FPy

**Fig. 6:**
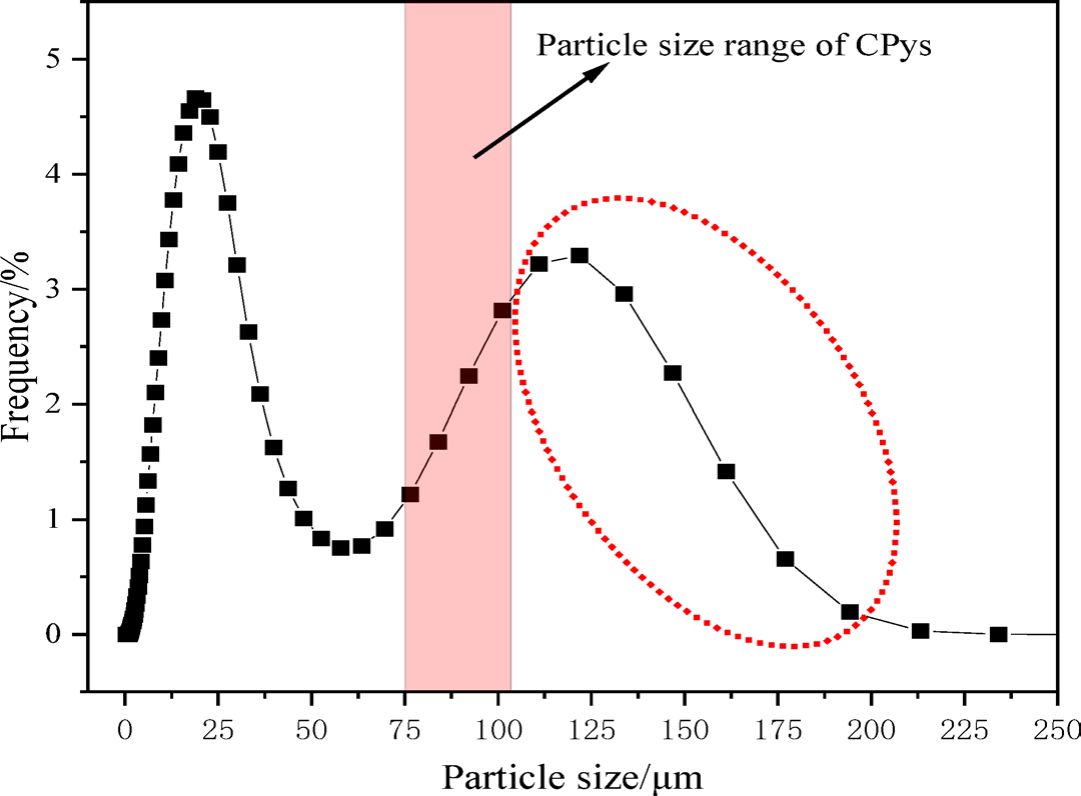
Particle size distribution of the mixture of CPys and FPys.

The effects of CPy on the recovery of FPy during flotation were primarily discussed in the previous section. It was hypothesized that the higher flotation recovery of the CPy and FPy mixture compared to the weighted value was due to the adhesion of FPy onto CPy. To confirm this hypothesis, a laser particle size analyzer was employed to analyze the particle size distribution of the CPy and FPy mixture. The mass ratio of CPy to FPy in the mixture was set at 1:1 to maintain consistency with the study conducted in “Flotation” section. The test results are presented in Fig. 6. The red area in Fig. 6 represents the particle size range of CPy (− 0.109 mm to + 0.075 mm). The observed particle size distribution of the CPy–FPy mixture deviated from the expected cumulative distribution of the individual components, suggesting particle agglomeration likely occurred between coarse and fine pyrite^56^. As shown in Fig. 6, the apparent particle size of the CPy and FPy mixture exceeded the maximum particle size of CPy (0.109 mm), as indicated by the red circle. This phenomenon suggests that the actual apparent particle size of pyrite in the mixture has increased, primarily due to significant agglomeration between CPy and FPy particles, although the possibility of agglomeration among CPy particles and among FPy particles cannot be excluded.

#### Observation of pyrite particle agglomeration

**Fig. 7:**
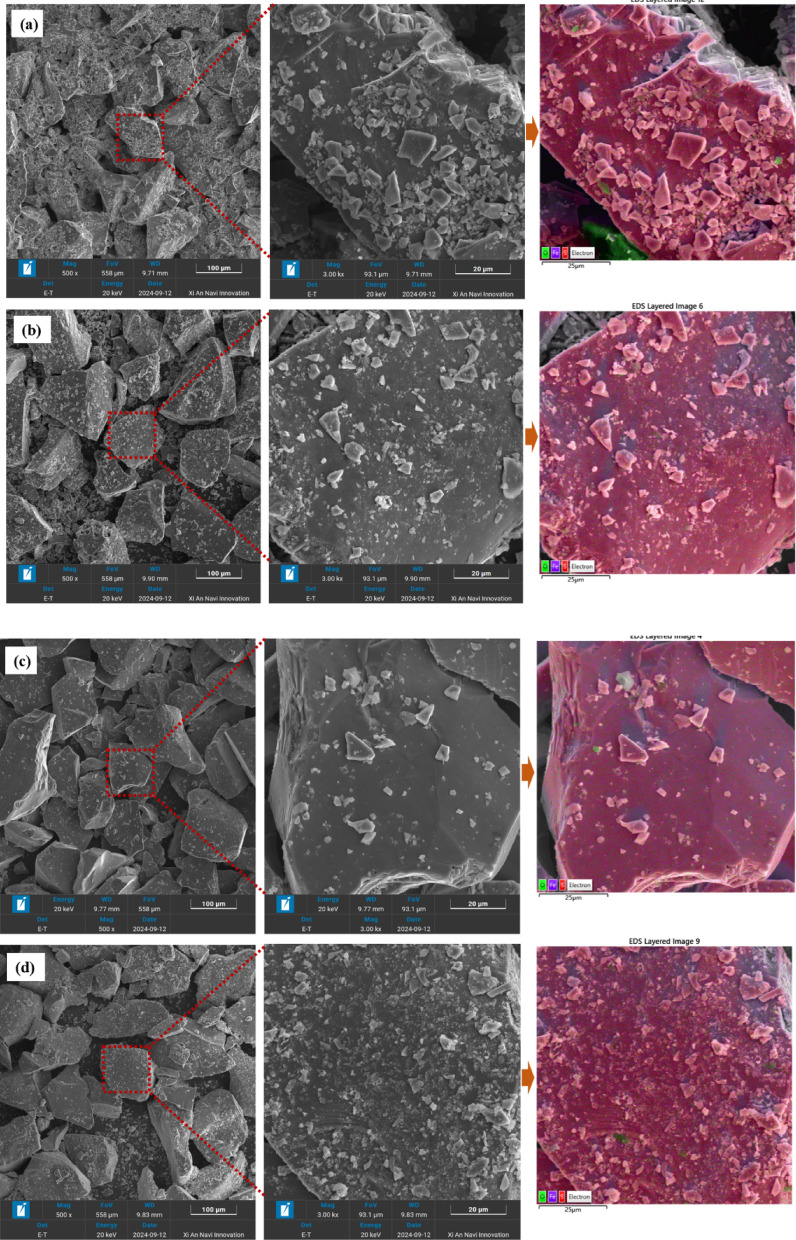
SEM images of the mixture of CPys and FPys in the presence or absence of butyl xanthate in the flotation cell ((**a**): in the absence of butyl xanthate at pH = 5; (**b**): in the presence of butyl xanthate atpH = 5; (**c**): in the absence of butyl xanthate at pH = 9; (**d**): in the presence of butyl xanthate at pH = 9).

According to the particle size distribution results in “Particle size analysis of mixture of CPy and FPy” section, FPy was likely to adhere to the surface of CPy. To verify this, SEM observations were conducted on CPy particles from mixed pulp samples (mass ratio of CPy to FPy = 1:1) in the presence and absence of butyl xanthate. It was worth noting that in this experiment, pH 5 was chose as the representative of acidic conditions and pH 9 was chose as the representative of alkaline conditions to further clarify the influence of acidic and alkaline conditions on the interaction behavior between CPy and FPy. The results are shown in Fig. 7.

FPy particles were observed to adhere to the surface of CPy under all tested conditions, but the degree of attachment varied. A greater amount of FPy was attached to CPy surfaces at pH 5, regardless of whether butyl xanthate was present, indicating that pyrite exhibited stronger hydrophobicity and more significant hydrophobic aggregation under acidic conditions. The particles easily adhered to each other by the hydrophobic force, which actually increased the roughness of the surface of the CPy. The improvement of this roughness helped to improve the hydrophobicity of the hydrophobic particles, thereby increasing the attachment between the particles and the bubbles, and this conclusion was discussed in the other two papers finished by the authors^57,58^. However, the influence of butyl xanthate was more significant at pH 9. In the absence of butyl xanthate, there was less FPy on the surface of CPy, likely due to the formation of hydrophilic substances on the surface of pyrite at this pH, which decreased the hydrophobic attraction between the mineral particles and reduced the attachment of FPy to the surface of CPy. Conversely, butyl xanthate interacted with the surface of pyrite, enhancing its hydrophobic attraction in the presence of the collector, thereby increasing the attachment of FPy to the surface of CPy. These observations confirm that FPy particles adhere to CPy surfaces under both acidic and alkaline conditions when butyl xanthate is present, indicating that collector-induced hydrophobic interactions facilitate particle aggregation.

**Fig. 8:**
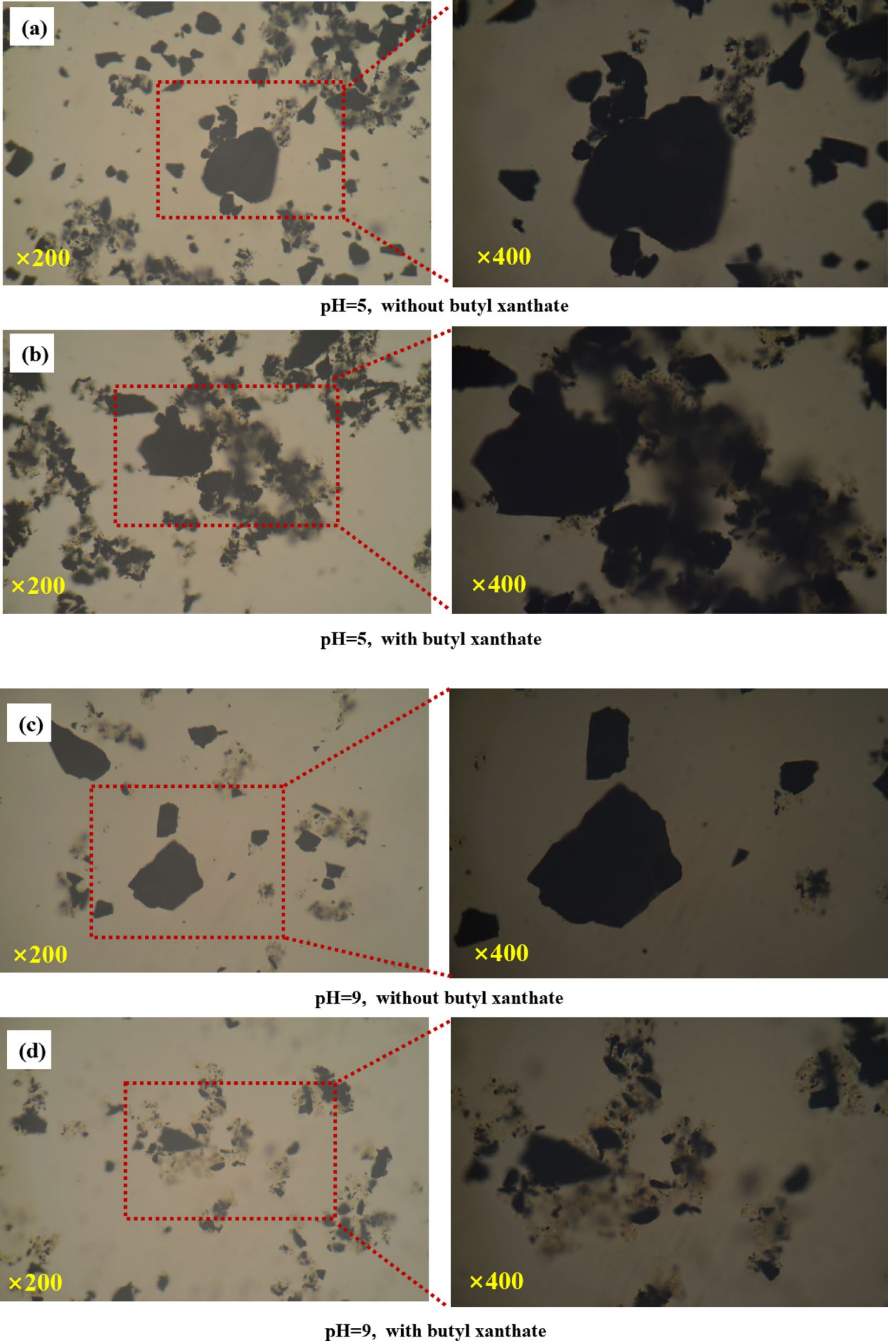
Micromorphology of of the mixture of CPys and FPys in the presence or absence of butyl xanthate in the flotation cell ((**a**): in the absence of butyl xanthate at pH = 5; (**b**): in the presence of butyl xanthate atpH = 5; (**c**): in the absence of butyl xanthate at pH = 9; (**d**): in the presence of butyl xanthate at pH = 9).

In addition, optical microscopy was employed to observe the aggregation behavior of CPy and FPy in mixed pulp under the same experimental conditions, further supporting the above conclusions. The morphology of the particles in the mixed pulp (with a mass ratio of CPy to FPy of 1:1) with and without butyl xanthate (concentration: 1 × 10^−3^ mol/L) at pulp pH 5 and 9 is shown in Fig. 8. At pH 5, a significant amount of FPy surrounded CPy particles, regardless of the presence of butyl xanthate, indicating strong interparticle adhesion (see Fig. 8a, b). In the absence of butyl xanthate at pH 9, CPy and FPy remained well-dispersed with limited agglomeration (Fig. 8c); however, when butyl xanthate was present, pronounced aggregation between CPy and FPy was observed (Fig. 8d). These results further suggest that butyl xanthate plays a critical role in promoting pyrite particle aggregation under alkaline conditions.

#### Observation of pyrite particle adhesion

**Fig. 9:**
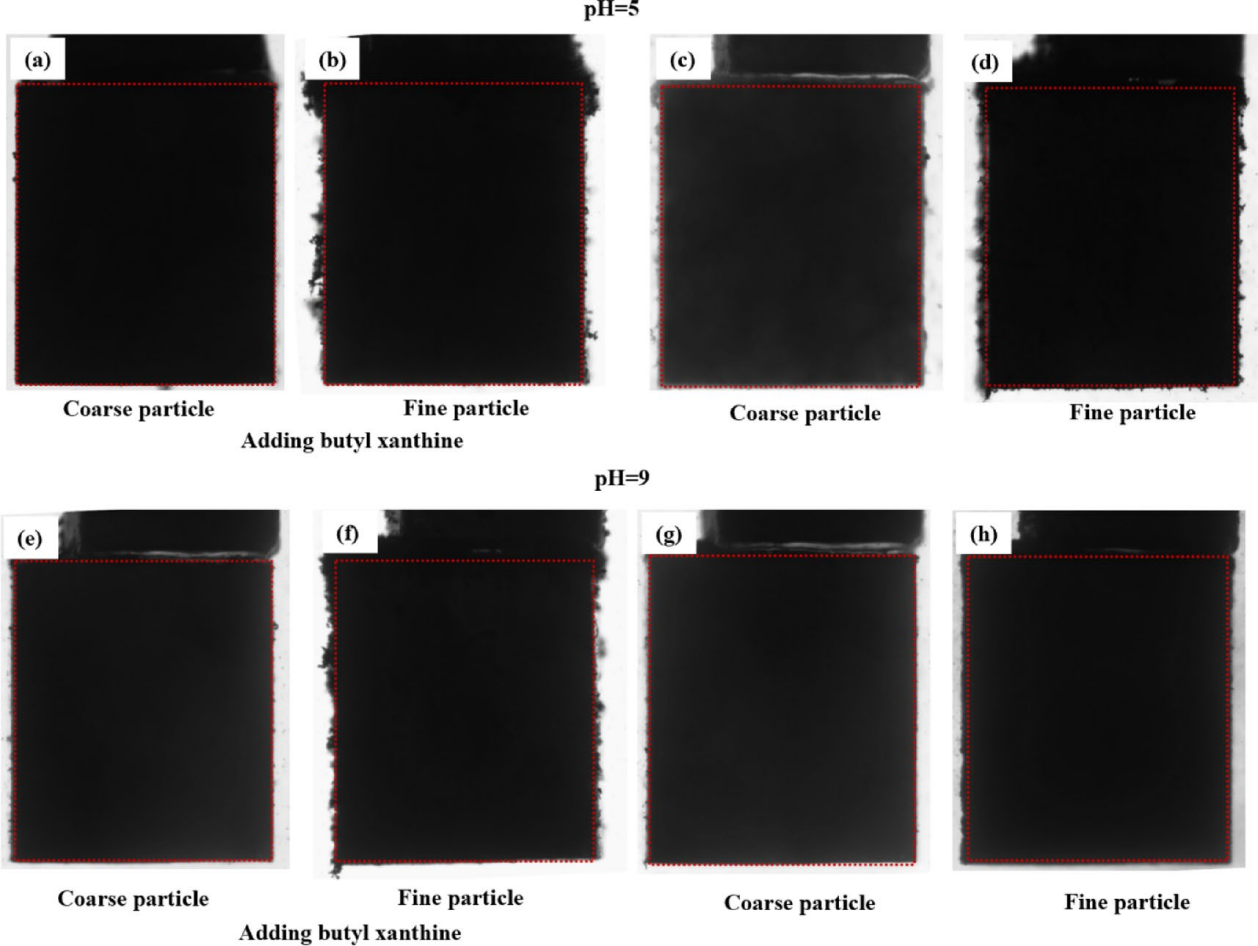
The morphology pictures of pyrite particles adhering on the surface of massive pyrite at pH 5 and 9 with and without butyl xanthine (1 × 10^–3^ mol/L) with a stirring time of 180 s.

While SEM and optical microscopy revealed the attachment of FPy to CPy surfaces under static conditions, they do not capture dynamic interactions. In this study, a self-made particle adhesion dynamic observation system was used to visualize and analyze the real-time attachment behavior of FPy to CPy surfaces under simulated flotation conditions. The snapshots (taken at 180 s) of pyrite particle attachment to the massive pyrite surface at pH 5 and pH 9 both in the presence and absence of butyl xanthate (1 × 10^−3^ mol/L) were shown in Fig. 9. As expected, the dynamic observations were consistent with the SEM and optical microscopy results. At pH 5, FPy adhesion to massive pyrite was slightly enhanced by the presence of butyl xanthate, as shown in Fig. 9b and d. However, a much more pronounced difference was observed at pH 9. as illustrated in Fig. 9f and h. In the presence of butyl xanthate at pH 9, the attachment of FPy to the surface of massive pyrite was significant, resulting in a “sawtooth” surface appearance in the projected two-dimensional image, indicating a substantial quantity of FPy on the massive pyrite. Conversely, the amount of FPy attached to massive pyrite was minimal in the absence of butyl xanthate at this pH, consistent with the results from SEM and optical microscopy, indicating that the hydrophobic force of pyrite was very weak under these conditions.

Notably, the adhesion of CPy particles to the surface of massive pyrite was significantly lower than that of FPy, highlighting the critical influence of particle size on adhesion behavior. Due to their large mass, coarse particles experience relatively weak hydrophobic forces compared to the gravitational and inertial forces acting upon them in the turbulent environment of a flotation cell. Consequently, particles are primarily influenced by gravity and inertia, making it challenging for CPy to adhere to the surface of massive pyrite under turbulent conditions. This finding further reinforces the conclusion that FPy are more likely to attach to CPy surfaces than CPy particles are to adhere to each other.

### EDLVO theoretical calculation

To gain deeper insight into the mechanism by which FPy adhere to the surface of CPy in suspension, the interparticle interaction energy was calculated based on the extended Derjaguin–Landau–Verwey–Overbeek (EDLVO) theory. According to EDLVO theory, the total interaction energy between particles consists of van der Waals forces, Electrostatic forces, and Hydrophobic forces. Attraction between particles predominates when the sum of these interaction forces is negative, and the repulsive forces are insufficient to overcome the attraction, leading to a reduction in the distance between particles and potentially resulting in aggregation or agglomeration. Specifically, van der Waals forces consistently act as attractive forces that promote particle aggregation. Electrostatic forces may be either attractive or repulsive, depending on the charge nature and density of the particles. Hydrophobic forces typically manifest as attractive forces, particularly between hydrophobic particles.

**Fig. 10:**
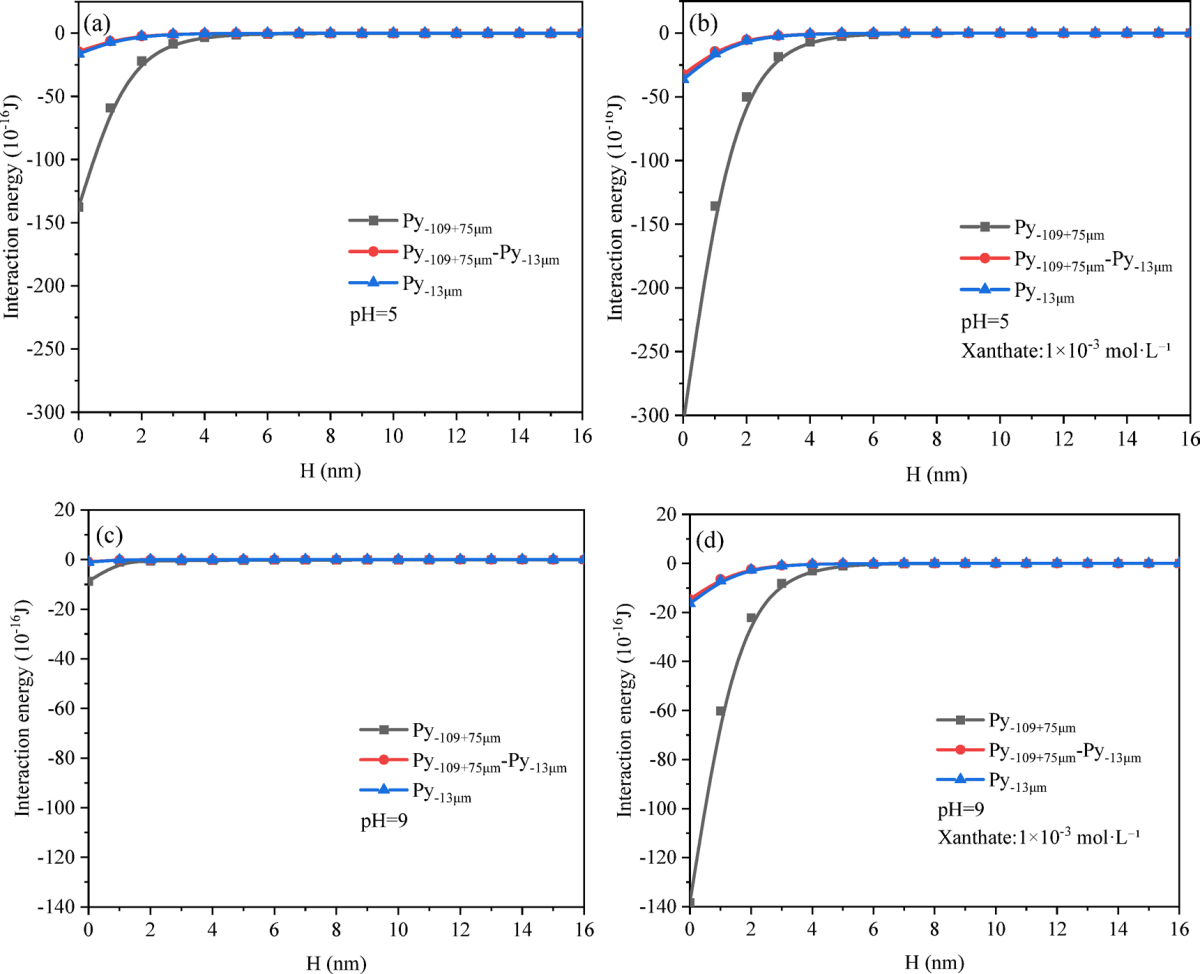
Interaction energy between CPy and CPy, CPy and FPy, FPy and FPy: (**a**) in the absence of butyl xanthate at pH 5; (**b**) in the presence of butyl xanthate at pH 5; and (**c**) in the absence of butyl xanthate at pH 9; (**d**) in the presence of butyl xanthate at pH 9.

In this study, interaction energies between particle pairs (CPy–CPy, CPy–FPy, and FPy–FPy) were calculated under various conditions (pH 5 and pH 9), with and without the presence of butyl xanthate. The zeta potential values of pyrite surfaces in deionized water and butyl xanthate solutions at pH 5 and pH 9 were measured using a Nano Sizer and Zeta-potential Tester (Zetasizer Nano ZS, Malvern, United Kingdom), and the results are shown in Table 4. Additionally, the isoelectric points of pyrite particles in deionized water and butyl xanthate solutions were determined as pH 5.82 and pH 2.97, respectively. Due to space constraints in this paper, the schematic diagrams of van der Waals energy, Electrostatic energy, and Hydrophobic energy are included in Figs. S-1, S-2, and S-3, respectively, in supplementary materials; instead, only the schematic diagrams of total energy versus distance for CPy and CPy, CPy and FPy, and FPy and FPy are provided in Fig. 10.

**Table 4:** The zeta potentials of pyrite.

Condition		Zeta potential/mv
pH = 9	Without butyl xanthate	− 7.03
	With 1 × 10^–3^ mol/L butyl xanthate	− 10.67
pH = 5	Without butyl xanthate	5.11
	With 1 × 10^–3^ mol/L butyl xanthate	− 10.23

As shown in Fig. 10a, the total interaction energy (VTED) between CPy–CPy, CPy–FPy, and FPy–FPy was negative at pH 5 in the absence of butyl xanthate, indicating the presence of net attractive forces among these particles, which could promote aggregation in the suspension under these conditions. Notably, the VTED values for CPy–FPy and FPy–FPy were similar at the same separation distance (H), suggesting comparable probabilities of aggregation between these particle pairs. The energy difference between CPy and CPy compared to CPy and FPy was substantial, and this difference in V_TED_ increased as H decreased when H was less than 3 nm, indicating that aggregation was more likely to occur between CPy and CPy than between CPy and FPy.

A similar trend is evident in Fig. 10b, which illustrates VTED profiles at pH 5 with the addition of butyl xanthate. The V_TED_ values for each particle in this condition were greater than those in the absence of butyl xanthate at the same H when H was less than 5 nm, indicating that butyl xanthate facilitated aggregation between pyrite particles. This was primarily attributed to the hydrophobic surface characteristics of pyrite particles induced by butyl xanthate, which enhanced hydrophobic attraction.

As shown in Fig. 10c, under alkaline conditions (pH 9), the VTED values for CPy–CPy, CPy–FPy, and FPy–FPy were all slightly negative, indicating weak but still attractive interactions among the particles. The V_TED_ values were larger than those in the system without butyl xanthate at the same distance H, attributed to the increased hydrophobic attraction between particles due to butyl xanthate. Therefore, butyl xanthate promotes aggregation between coarse and fine pyrite particles in suspension under both acidic and alkaline conditions. However, the magnitude of VTED under alkaline conditions was approximately half that under acidic conditions, indicating that pyrite aggregation is more favorable in acidic environments. These theoretical results are in good agreement with the experimental findings.

## Conclusions

Pyrite from a gold ore in southwestern Guizhou Province, China, was selected as the sample to investigate the interaction behavior between CPy and FPy and its effect on flotation recovery. The adhesion behavior of FPy on CPy was examined using the Particle Attachment Dynamic Observation System, along with a laser particle size analyzer, optical microscopy, SEM, and EDS. The mechanisms of agglomeration between FPy and CPy were further analyzed by EDLVO theoretical calculations. The following conclusions were drawn:

1. The flotation recovery of pyrite exhibited a trend of first increasing and then decreasing with increasing dosages of the collector (butyl xanthate), with an optimal dosage identified at 1 × 10⁻^3^ mol/L. Under the same collector concentration, the flotation recovery of FPy (P_80_ = 26.75 µm) was lower than that of CPy (P_80_ = 147.89 µm). However, the recovery of the CPy–FPy mixture exceeded that of either component alone, particularly at low collector dosages, indicating a synergistic effect due to particle interactions.
2. Laser particle size analysis revealed that the apparent particle size of the CPy–FPy mixture exceeded the maximum particle size of CPy alone, implying agglomeration between CPy and FPy. SEM, optical microscopy, and the Particle Attachment Dynamic Observation System confirmed that significant FPy adhered to CPy at pH 5, both with and without butyl xanthate. In contrast, at pH 9 without butyl xanthate, CPy and FPy were mostly dispersed in the pulp. However, the addition of butyl xanthate promoted FPy adhesion to CPy at pH 9, indicating that butyl xanthate is a key factor facilitating agglomeration under alkaline conditions.
3. EDLVO calculations showed net attractive interactions between particles at both pH 5 and pH 9, regardless of the presence of butyl xanthate, although the magnitude of attraction varied with pH, collector addition, and particle size. The weakest attraction occurred at pH 9 without butyl xanthate. The presence of butyl xanthate significantly enhanced the hydrophobicity of pyrite particles under both acidic and alkaline conditions, which was the primary driver of pyrite agglomeration. This enhanced hydrophobic attraction also explains the improved flotation recovery observed for the CPy–FPy mixture compared to individual components in the presence of butyl xanthate.

## Electronic supplementary material

Below is the link to the electronic supplementary material.

Supplementary Material 1

## Data Availability

The datasets used and/or analysed during the current study available from the corresponding author on reasonable request.
