# Supplementary material for: Interaction behavior between coarse-particle pyrite and fine-particle pyrite in flotation
Source: Sci Rep. 2025 Jul 2;15:22500. doi: 10.1038/s41598-025-06128-0 (PMC12217499; doi:10.1038/s41598-025-06128-0)
Supplement: Supplementary file 1 — Supplementary Material 1 [file 41598_2025_6128_MOESM1_ESM.docx]

Fig. S-1. Van der Waals energy between CPy and CPy, CPy and FPy, FPy and FPy: (a) in the absence of butyl xanthate at pH 5; (b) in the presence of butyl xanthate at pH 5; and (c) in the absence of butyl xanthate at pH 9; (d) in the presence of butyl xanthate at pH 9.

Fig. S-2. Electrostatic energy between CPy and CPy, CPy and FPy, FPy and FPy: (a) in the absence of butyl xanthate at pH 5; (b) in the presence of butyl xanthate at pH 5; and (c) in the absence of butyl xanthate at pH 9; (d) in the presence of butyl xanthate at pH 9.

Fig. S-3. Hydrophobic energy between CPy and CPy, CPy and FPy, FPy and FPy: (a) in the absence of butyl xanthate at pH 5; (b) in the presence of butyl xanthate at pH 5; and (c) in the absence of butyl xanthate at pH 9; (d) in the presence of butyl xanthate at pH 9.
